# Pattern of Visits in a Metropolitan Emergency Department in Lombardia (Italy): January 2019–December 2020

**DOI:** 10.3390/healthcare9070791

**Published:** 2021-06-24

**Authors:** Simone Gambazza, Alessandro Galazzi, Filippo Binda, Onorina Passeri, Paola Bosco, Giorgio Costantino, Dario Laquintana

**Affiliations:** 1 Healthcare Professions Department, Fondazione IRCCS Ca’ Granda Ospedale Maggiore Policlinico, Via Francesco Sforza 35, 20122 Milano, Italy; simone.gambazza@policlinico.mi.it (S.G.); alessandro.galazzi@policlinico.mi.it (A.G.); onorina.passeri@policlinico.mi.it (O.P.); paola.bosco@policlinico.mi.it (P.B.); dario.laquintana@policlinico.mi.it (D.L.); 2 Emergency Department, Fondazione IRCCS Ca’ Granda Ospedale Maggiore Policlinico, Via Francesco Sforza 35, 20122 Milano, Italy; giorgio.costantino@unimi.it; 3 Department of Clinical Sciences and Community Health, University of Milan, Via Francesco Sforza 35, 20122 Milano, Italy

**Keywords:** COVID-19, Italy, emergency service, overcrowding, primary care

## Abstract

During the Coronavirus disease 2019 (COVID-19), a general decrease in the presentations to emergency departments (ED) was reported. However, we suspect that there was a lower number but an unchanged pattern of ED visits for urgent conditions in 2020 compared to 2019. This retrospective study assessed the change in the number of presentations in the ED of a tertiary level university hospital in Milano (Lombardia, Italy). Compared to 2019, a significant drop in ED presentations occurred (−46.4%), and we recorded a −15.7% difference in the proportion of patients admitted with white codes. The pattern of hourly presentations to the ED was unchanged, with overcrowding during the working daytime. COVID-19 changed ED flows, likely causing an overall reduction in the number of deferrable conditions. However, the pattern associated with urgent conditions did not change abruptly in 2020.

## 1. Introduction

Italy was the first European country involved in the Coronavirus disease 2019 (COVID-19) pandemic. The general attitude suggested by health authorities was postponing deferrable interventions, which lead to a drop in the number of people seeking hospital care. As reported elsewhere, people suffering from neurologic [1] and cardiac [2] diseases have vanished from emergency departments (EDs), feeding the suspicion of increased overall mortality as the most devastating consequences of the pandemic on the care of diseases other than COVID-19. For instance, it was estimated that the total number of deaths in December 2020 was over 25,000 in Italy [3]. Despite the fear of contagion and the general recommendation to avoid unnecessary visits to EDs, we hypothesized that a lower volume but unchanged pattern of ED visits for urgent conditions occurred in 2020 compared to 2019.

The aim of the present analysis is to investigate the pattern of presentations to the ED of Fondazione IRCCS Ca’ Granda Ospedale Maggiore Policlinico in Milano, a metropolitan ED of a tertiary level university hospital in Lombardia.

## 2. Materials and Methods

We retrospectively analyzed the number of all ED presentations during 2019 and 2020, together with demographic data and symptoms. We excluded women with obstetric or gynecologic conditions and patients aged <18 years who were managed in another dedicated ED within the same hospital. Data are expressed as absolute numbers and proportions or means and standard deviations. The main findings are presented as the difference between proportions or mean difference, with a 95% confidence interval (CI). The Wilcoxon Rank Sum test was used to test the difference between continuous data. *P*-values < 0.05 denote statistically significant results. Analyses were performed using R Core Team, version 4.0.3 [4].

## 3. Results

**Figure 1:**
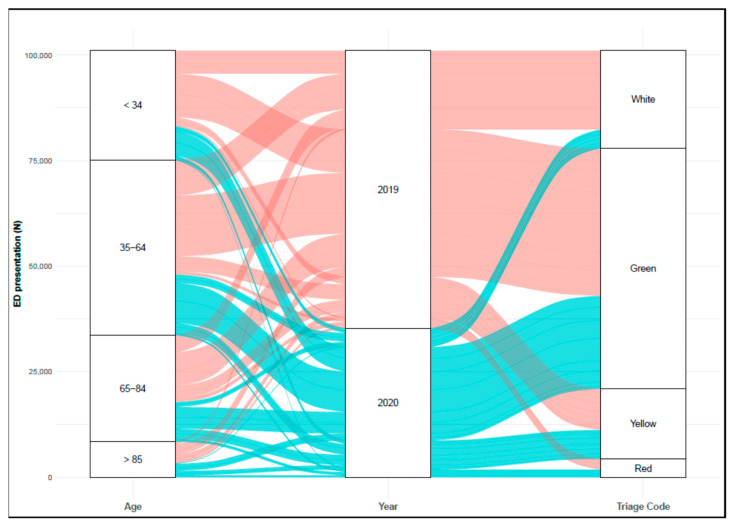
Alluvial plot for the number of ED presentations in 2019 and 2020. The height of the blocks represents the proportion of observations in that cluster, and the height of the stream field represents the proportion of observations contained in both blocks they connect. Stream field colors denote the years 2019 (pink) and 2020 (blue). The triage code denotes the classification of disease severity following the current Italian ED triage scoring system (white: no need for emergency treatment; green: need for fast treatment; yellow: severe condition; red: life-threatening condition).

There were 35,249 ED presentations in our hospital in 2020, compared to 65,804 in 2019 (Figure 1), which resulted in a reduction of 46.4%, and involved fewer females compared to the male population, 50.2% versus 42.5%, respectively. 

Overall, the number of resulting hospital admissions were 7281 (11.1%) in 2019 compared to 6353 (18.0%) in 2020 (*p* < 0.001). Furthermore, we reported more deaths in our ED (192 versus 285) in 2020, which corresponds to a 0.5% difference in mortality (95% CI: 0.4 to 0.6%). The population visiting the ED in the 2020 period was older, 54.8 (21.4) versus 52.2 (21.4) years in 2019. Only people with minor trauma, abdominal or chest pain, and ear-nose-throat-related conditions did show a statistically significant variation in age between 2019 and 2020. Mean difference in age was, respectively, 1.2 (95% CI:0.4 to 2.0), 1.5 (95% CI:0.6 to 2.5), 1.5 (95% CI:0.3 to 2.8), and 1.8 (95% CI:0.7 to 2.9) years.

The top-10 reported conditions or symptoms in patients referred to the ED in 2019 are displayed in Table 1.

Despite numbers being almost halved in 2020, it is interesting to note that the proportion of people presenting with chest pain was constant over time (i.e., 4% versus 3.9%), despite it affecting older individuals in 2020 compared to the previous year, +1.5 (95% CI: 0.3 to 2.8) years old. We also witnessed a lower proportion of people referred to ED for dermatologic and ophthalmologic conditions, ear-nose-throat-related issues, and single-arm pain. The number of people with dyspnea abruptly increased by 14.6%, without a clinically significant difference in age, −0.1 (95% CI −1.1 to 1.1) years. 

Compared to 2019, it is worth noting that we also recorded a 0.5% (95% CI: 0.4 to 0.6) and 0.1% (95% CI: −0.01 to 0.1) increase in the number of discharge and abandonment cases in people presented with dyspnea, respectively.

Among the priority codes of access (i.e., triage codes), we recorded a −15.7% difference in the proportion of patients admitted with white codes in 2020. On the contrary, green, yellow, and red codes showed a positive difference of 9.5%, 4.7%, and 1.5%, respectively. Presentations with major urgency (i.e., yellow) in 2020 showed the greatest difference in age, +3.2 (95% CI: 2.5 to 3.8) years compared to 2019. Non-urgent conditions (i.e., white and green codes) represented 90.6% and 89.5% of people with other symptoms both in 2019 and 2020.

**Figure 2:**
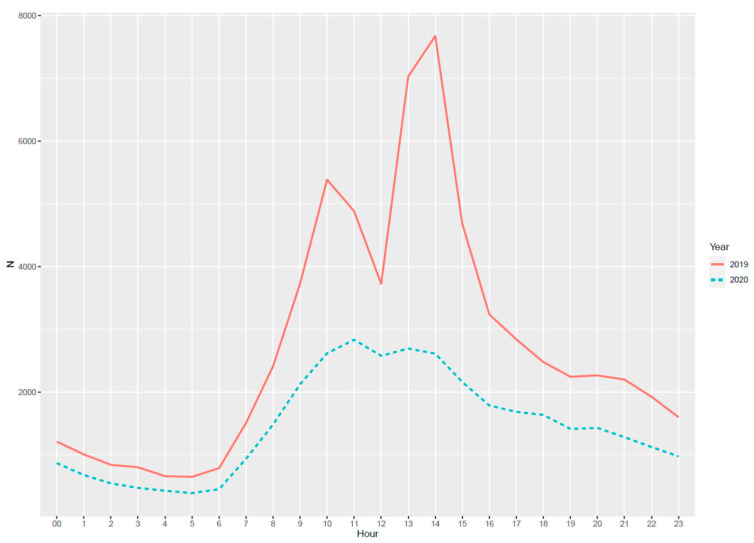
Hourly referrals to ED in 2019 and 2020. The bimodal distribution denotes a drop in the presentations due to shift change in the ED tracks dedicated to dermatologic-, ophthalmologic-, and ear-nose-throat-related conditions.

In terms of ED performance, overall median waiting time-to-visit decreased by 17 min (*p* < 0.001) in 2020 compared to the year before. If we consider the time of ED presentation during the whole day, we reported the same pattern between the observed years (Figure 2). 

## 4. Discussion

This retrospective analysis supports other groups’ findings reporting a reduction in patients’ accesses to the ED [5,6], however, we cannot claim that COVID-19 dramatically changed the pattern of urgent visits to our ED. Over recent years, we have seen the ED becoming multi-professional outpatient clinics, where patients ask for timely interventions in response to the difficulties of access to general practitioner (GP) primary care and advanced examinations, usually subjected to long waitlists. The ED is quicker than getting an appointment with the GP, and there is all the medical expertise required to solve the perceived acuity of the condition. 

Regarding the reduction in the number of ED presentations, it is likely that self-selection of inappropriate ED presentations occurred during the COVID-19 pandemic. Some people with deferrable conditions respected the *stay-home* recommendation for fear of contagion, and these might have presented with dermatologic, ophthalmologic, and single-arm pain-related issues. To this, we should also consider that some people decided to leave big cities and headed back to their native homes because of the pandemic, which exposed fewer people to noxious events. On the other hand, some others continued to seek medical attention, mostly because they felt it urgent. Indeed, minor traumatic injuries and pain were the most frequent reasons for showing up to the ED. Pain is strictly related to the perceived acuity and urgency of the condition, eliciting abnormal reactions at the emotional level, which requires immediate control. Media contributed to increasing the arousal level in the population about dyspnea, which was appointed as highly indicative for COVID-19. 

Independently from the pandemic, the bottom line is that people decided to spend hours waiting for a visit in the ED during working daytime rather than going to their GP, as depicted in Figure 2. In a metropolitan area like Milano, patients seeking emergency care should be expected during out-of-hours care when GP outpatients are closed. With a differential of 30,555 ED presentations in 2020, 88.6% were non-urgent visits. 

Being aware that we are offering a partial perspective of a more complex phenomenon and that we relied on reasons for ED presentation based on patients’ reported symptoms, we wonder what future is planned for our hospitals within the current regional health system. As reported by Morello et al., ED overuse and overcrowding are phenomena that are not affordable [7] and require specific interventions [8]. To date, several strategies are available to improve the flow in the emergency department, such as the use of validated scores and the clinical judgment of the triage nurse [9].

## 5. Conclusions

COVID-19 changed ED flows, likely causing an overall reduction in the number of deferrable conditions. However, the pattern for urgent conditions did not change abruptly in 2020. Potentiating territorial medicine and lower-acuity-care services would give back to EDs their role as hyper-specialistic places for urgent and emergency conditions rather than crowded outpatient services.

## Figures and Tables

**Table 1:** Top-10 reasons for ED presentations in 2019 compared to 2020.

	2019	2020	
	N (%)	N (%)	Difference [95%CI]
Dermatologic	12,348 (18.8)	2024 (5.7)	−13 [−13.4 to −12.6]
Minor trauma	8532 (13.0)	4643 (13.2)	0.2 [−0.2 to 0.6]
Abdominal pain	4864 (7.4)	2766 (7.8)	0.5 [0.1 to 0.8]
Other symptoms	3939 (6)	2708 (7.7)	1.7 [1.4 to 2.0]
Ear-nose-throat	3916 (5.9)	1743 (4.9)	−1 [−1.3 to −0.7]
Chest pain	2641 (4)	1376 (3.9)	−0.1 [−0.4 to 0.1]
Single-arm pain	2454 (3.7)	1184 (3.4)	−0.4 [−0.6 to −0.1]
Dyspnea	2343 (3.5)	2685 (7.6)	4.1 [3.7 to 4.4]
Eyes	2316 (3.5)	1114 (3.2)	−0.4 [−0.6 to −0.1]
Asthenia	1735 (2.6)	1098 (3.1)	0.5 [0.3 to 0.7]
Total	65,804	35,249	

## Data Availability

Data are available from the authors upon reasonable request and with permission of Fondazione IRCCS Ca’ Granda Ospedale Maggiore Policlinico.
